# Eco-Friendly Recycling of Lithium Batteries for Extraction of High-Purity Metals

**DOI:** 10.3390/ma16134662

**Published:** 2023-06-28

**Authors:** Gamal M. A. Mahran, Mohamed A. Gado, Wael M. Fathy, Amr B. ElDeeb

**Affiliations:** 1 Mining Engineering Department, King Abdulaziz University, Jeddah 21589, Saudi Arabia; gmahran@kau.edu.sa; 2 Nuclear Materials Authority, Maadi, Cairo 530, Egypt; 3 Mining and Petroleum Department, Faculty of Engineering, Al-Azhar University, Cairo 11884, Egypt; wael_fathy@egyptire.com (W.M.F.); dr.basuony2016@azhar.edu.eg (A.B.E.)

**Keywords:** spent lithium-ion batteries, lithium, cobalt, extraction, hydrometallurgy, adsorbent, adsorption, kinetics

## Abstract

The significant increase in lithium batteries consumption produces a significant quantity of discarded lithium-ion batteries (LIBs). On the one hand, the shortage of high-grade ores leads to the necessity of processing low-grade ores, which contain a low percentage of valuable metals in comparison to the discarded LIBs that contain a high percentage of these metals, which enhances the processing of the discarded LIBs. On the other hand, the processing of discarded LIBs reduces the negative environmental effects that result from their storage and the harmful elements contained in their composition. Hence, the current study aims at developing cost-effective and ecofriendly technology for cobalt and lithium metal ion recovery based on discarded LIBs. A novel synthesized solid-phase adsorbent (TZAB) was utilized for the selective removal of cobalt from synthetic solutions and spent LIBs. The synthesized TZAB adsorbent was characterized by using 13C-NMR, GC-MS, FT-IR, 1H-NMR, and TGA. The factors affecting the adsorption of cobalt and lithium ions from synthetic solutions and spent LIBs, including the sorbent dose, pH, contact time, temperature, and cobalt concentration were investigated. The conditions surrounding the recovery of cobalt and lithium from processing discarded LIBs, were investigated to optimize the maximum recovery. The Langmuir, Freundlich, and Dubinin–Radushkevich (D-R) isotherm models were used to study the kinetics of the adsorption process. The obtained results showed that high-purity CoC_2_O_4_ and Li_3_PO_4_ were obtained with a purity of 95% and 98.3% and a percent recovery of 93.48% and 95.76%, respectively. The maximum recovery of Co(II) from synthetic solutions was obtained at C_0_ = 500 mg·L^−1^, dose of 0.08 g, pH 7.5, T = 25 °C, and reaction time = 90 min. The collected data from Langmuir’s isotherm and the adsorption processes of Co agree with the data predicted by the D-R isotherm models, which shows that the adsorption of Co(II) onto the TZAB seems to be chemisorption, and the results agree with the Langmuir and D-R isotherm models.

## 1. Introduction

Electronic products, particularly mobile ones such as cell phones and laptops, have shorter lifespans than they used to because of the fast rate at which new technologies are developed. Because of this, there has been a growth in the quantity of used electronic and electrical equipment, such as LIBs [1,2]. Since these batteries contain harmful materials such as heavy metals and electrolytes, their disposal can have negative consequences for the environment. These important metal ions include cobalt and lithium, which are crucial and appreciated metals, and the recycling of them is essential [3].

Recycling LIBs not only helps prevent pollution, but it also results in the better use of scarce materials and may reduce the cost of producing new batteries. In addition, high-purity metals can be recovered from these wastes, and in some situations, the concentration of these interesting metals in batteries is much higher than their content in their ores [4,5]. Nowadays, the rising cost of cobalt production has made cobalt recycling a reasonable option [6]. About a quarter of the world’s cobalt consumption in 2007 was for the manufacturing of batteries [7].

The model solution containing Co^2+^ was chosen as it represents a common contaminant in industrial wastewater that is generated from the recycling of lithium-ion batteries, where cobalt is a key component. Cobalt is known to form stable complexes with various ligands, making it a suitable analyte to test the performance of the newly developed adsorbent material.

Cobalt (Co) is a transition metal that is employed in a wide-ranging variety of contexts, including but not limited to the mining, catalyst, electronic, alloy, pigment, agricultural, and steel industries, as well as in systems of biology, where it is required to produce vitamin B12 [8]. Activation yields the element cobalt-60 (^60^Co), which finds uses in medical applications, medical device sterilization, and food preservation [9]. Cobalt has been detected in sediment and water bodies [10,11] due to its numerous applications, the formation of vast quantities of waste with poor or no management, and its great concentrations.

There can be physicochemical interactions between industrial or nuclear wastes of cobalt, spent lithium cobalt batteries that have been disposed of without pretreatment, and the organic (fulvic acids and humic acid) and mineral constituents of soil, leading to procedures such as sorption, ion exchange, or the formation of complexes. Overexposure to cobalt has negative consequences for human and environmental health [12]. Chemical precipitation, filtration, ion exchange, reverse osmosis, and adsorption are just a few of the methods recommended to remove cobalt in water [13,14].

A mass transfer phenomenon, adsorption involves the uptake of a material (the adsorbate) from a gas or liquid by a material in the solid phase (the adsorbent). The high removal efficiency, straightforward application, and the availability of low-cost adsorbents have made this method the preferred method for pollution treatment. Activated carbon [15], chitosan [16], carbon nanotubes and alumina [12], palygorskite [17], sepiolite [18], resins [19], chitin [20], soils, and biosorbents such as almond green hull [21], watermelon rind [22], rice straw [23], spirulina sp. algae [24], and shewanella spp. are used in this process. Because of their portability, high number of charge/discharge cycles, and low memory effect, LIBs have become the most popular type of rechargeable battery [25].

Compared to solvent extraction, adsorption is significant for a number of reasons: Adsorption is a cheaper alternative to solvent extraction. Adsorption materials are typically less expensive than solvents, and the procedure requires less equipment and energy. Adsorption is a technique that is less harmful to the environment than solvent extraction. Using hazardous solvents to extract solvents is a common practice that endangers both the environment and human health. Adsorption makes use of organic substances that are risk free and nontoxic. In comparison to solvent extraction, adsorption is more effective. Additionally, the increased surface area of adsorption materials allows them to adsorb more of the target ingredient than solvents can. Additionally, whereas solvent extraction is restricted to a specific set of solvents and chemicals, adsorption can be used to extract a large range of substances from a variety of solutions. Overall, adsorption is a more practical and efficient technique than solvent extraction for many purposes [26,27].

LIBs are widely employed in imported electronic and informational gadgets, including mobile phones, laptops, and other portable computing and communication equipment. The automotive sector, including hybrid and electric vehicles, and large-scale grid energy storage have both benefited from the spread of the green energy concept and the incorporation of alternative energy sources [28,29]. Between 2014 and 2019, the utilization of LIBs in portable electronics increased, with 29% of all batteries being lithium nickel manganese cobalt oxide batteries and 37% of all batteries being lithium cobalt oxide (LCO) batteries [30,31]. Only the mobile phone and computer industries account for 33.1 billion USD of the total market value of LIBs [32].

Nearly 90% of the value of used LIBs comes from the metals contained within them. The active cathode layer contains these metals. When it comes to the business of managing and recycling LIBs, cobalt is by far the most valuable component and the most lucrative commodity traded. It is anticipated that 4500 tons of cobalt are present in 1,200,000,000 cell phone batteries with an average weight of 20 g/unit [33,34]. There is an estimated 8900 USD per ton market for the recycling of lithium cobalt oxide batteries, while recycling lithium manganese oxide batteries is seen as unprofitable and inconvenient, with a capacity of just 860 USD per ton. Cobalt’s 2017 economic value per ton of waste LIBs was projected to be 55,000 USD. The European Commission has classified cobalt as a critical, precious, and strategic metal due to its fixed production in 2017, posing a threat to the market and supply chain (EU, 2017). More than fifty percent of the world’s cobalt funds will be depleted by the LIBs industry by 2025 [35,36].

The current study aims at exploring the preparation and characterization of a novel synthesized adsorbent for the sorption of high-purity cobalt and lithium based on spent LIBs through adsorption by using a modified triazole Schiff base. The factors affecting the sorption of cobalt ions, including pH, sorbent dose, contact time, temperature, and cobalt concentrations, were investigated. Additionally, the kinetics of the adsorption processes were studied.

## 2. Materials and Methods

### 2.1. Materials

All the chemicals used in the current study were of analytical grade and obtained from Sigma-Aldrich (St. Louis, MO, USA) and Merck (Darmstadt, Germany). The synthetic reactions involving metal complexes and the ligand were performed by using solvents that were distilled and dried, as is customary for such techniques in the literature. The decomposition temperatures of the metal complexes and the melting points of the produced ligands were determined with a Stuart melting point instrument.

Using a Nicolet FT-IR Impact 400D spectrometer, infrared spectra of the solids (by using a KBr matrix) in the 3700–370 cm^−1^ area were recorded. The Bruker Advance 300 MHz equipment was used to obtain the 1H and 13CNMR spectra. The ligand mass spectra were captured with a JEOL MS Route instrument. At room temperature, magnetic susceptibility was measured by weighing the complexes against a standard of mercury acetate ligand in a Stanton SM12/S Gouy balance.

### 2.2. Methods

#### 2.2.1. Preparation of the Aqueous Solutions of Cobalt

Cobalt (II) nitrate hexahydrate (Co(NO_3_)2.6H_2_O) (J. T. Baker, 99%, Phillipsburg, NJ, USA) and deionized (DI) water were used to prepare all the aqueous solutions. Except for the experiments conducted to test the impact of pH upon sorption, all the cobalt solutions were adjusted at a pH of 5.5. Atomic absorption spectrophotometry (AAS) (Varian AA240) was used to analyze the cobalt content, with the calibration curve established with a cobalt standard (Merck) of 1000 mg/L.

#### 2.2.2. Adsorption via Batch Technique

The batch adsorption mode tests were accomplished by reserving the solution in conjunction with a proper dose of PST-SA in an Erlenmeyer flask (100 mL) in a shaker incubator of the type FTSH-301 MINI (SP) at 150 rpm. Each experiment was conducted to determine the Co^+2^ adsorption by utilizing 0.1 g/L PST-SA at pH values between 2 and 10, with an initial Co^+2^ concentration of 250 mg/L. The mixture was shaken for 30 min at 25 °C before starting the experimental studies on the adsorption process.

NaOH and HCl solutions were utilized for pH regulation. To determine the optimum adsorbent dose, a set of examinations was conducted by utilizing a dosage between 0.01 and 0.25 g/L at the optimum pH value. No novel parameters of operation were introduced in this set of evaluations. The kinetic experiments were conducted at 25 °C at a 250 mg/L initial concentration and for times ranging from 5 to 120 min. The initial Co^+2^ concentrations in the equilibrium experiments varied from 250 to 1500 mg/L. The time of equilibrium was calculated via adsorption kinetics.

The sorbents’ abilities to absorb Co^+2^ ions in the presence of another interfering ion were measured with several binary adsorption tests. For the multicomponent adsorption experiments, the ending Co^+2^ concentrations were determined by using UV-VIS spectroscopy (Rigol-Ultra-3660 spectrophotometer) and atomic absorption spectroscopy (by using a Varian AA240). A duplicate observation of each experiment was performed, and the mean values were reported. The adsorption capacity qt (mg/g) was calculated according to Equations (1) and (2).

q_t_ = (C_o_ − C_t_)V)/m (1)

S% = (C_o_ − C_e_)/C_o_ (2)

where q_t_ is the concentration of the adsorbed cobalt ions per unit mass at time t (mg/g); qe is the equilibrium concentration (mg/g); C_o_ is the initial concentration of cobalt ions in the solution (mg/mL); C_e_ and C_t_ is the cobalt ion concentration at time t or at equilibrium (mg/mL), respectively; V is the aqueous solution volume containing cobalt ions (L); and m is the adsorbent mass (g).

#### 2.2.3. Preparation of the Adsorbent

**Scheme 1:**
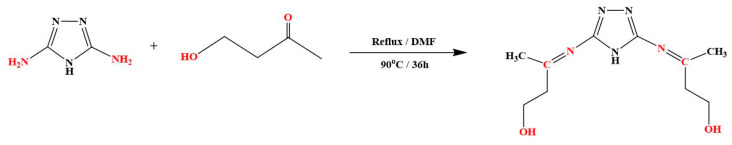
A novel triazole Schiff base was created by condensing 3,5-diamino-1,2,4-triazole and 4-hydroxybutan-2-one in a 1:2 mass ratio by using alkali-fusion method.

In order to prepare the adsorbent, 2.0 g of 3,5-diamino-1,2,4-triazole corresponding to (10.0 mmol), in addition to 9.90 g 4-hydroxy 2-butanone corresponding to (20.0 mmol), were mixed by using a three-neck round bottom flask (250 mL has 3 arms) sited on a hot plate with a stirrer associated with a condenser and temperature controller. A total of 25 mL of di-methylformamide (DMF) was utilized as the solvent of the reaction. The reaction mix was refluxed for 36 h at 90 °C, as presented in Scheme 1. After the completion of the reaction, the remaining solvent was evaporated by using an evaporator. To eliminate all traces of the solvent, the resultant pale-yellow product was dried at 50 °C for 6 h by using a vacuum oven before being measured. It was noted that the final yield was 10.7 g, which represents about 92.68%. This chemical is microcrystalline solid in nature, has a 280 °C melting point, and is pale yellow in color. At room temperature, this ligand was soluble in DMF and DMSO.

#### 2.2.4. Adsorbent Characterization

Inductively coupled plasma optical emission spectroscopy (ICP-OES) (Optima 7000DV, PerkinElmer, MA, USA) was utilized to detect the concentrations of metal ions in a given solution. Thermo Fischer Scientific’s Nicolet™ iS10 spectrophotometer (Nicolet iS10, Morris Plains, NJ, USA) was used to carry out the FTIR spectra. 1H (CD4O, 500 MHz) and (CD4O, 202 MHz) nuclear magnetic resonance (NMR) spectra of IL and IL-ICR (IL after extraction) were documented on a Bruker 500 MHz NMR spectrometer (AVANCE III HD 500 MHz, Bruker BioSpin GmbH, Ettlingen, Germany). The structure and morphology of the prepared adsorbent were characterized by using an energy-dispersive X-ray spectrometer attached to a field emission scanning electron microscope (SEM, Hitachi S-4800, Tokyo, Japan) (EDS, Genesis XM2, EDAX, Pleasanton, CA, USA).

## 3. Result and Discussion

### 3.1. Characterization of TZAB

**Figure 1:**
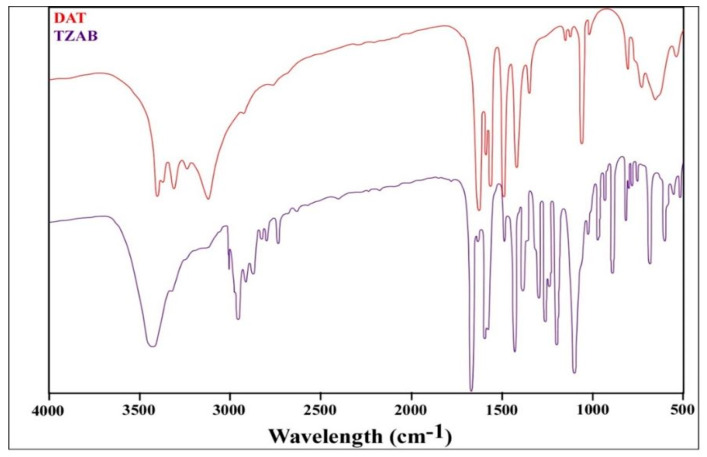
FT-IR spectra of DAT and triazole Schiff base (TZAB).

Firstly, the molecular structure of the produced adsorbent was characterized by using FT-IR spectroscopy. A comparison of the infrared spectra of 3,5-diamino-1,2,4-triazole (DAT) and the triazole Schiff base (TZAB) is presented in Figure 1. The symmetric and asymmetric N-H stretching of the primary amine groups linked to C3 and C5 of the triazole ring was responsible for the two absorption bands observed at 3400 and 3100 cm^−1^ in the triazole spectra, respectively [37]. The N-H stretching peak at 3300 cm^−1^ could be traced back to the secondary -NH group present in the triazole ring. N-H bending (-NH_2_) and C=N stretching (ring) were responsible for the intense absorption peaks at 1635 and 1554 cm^−1^, respectively. The C-N stretching (triazole ring) exhibited a modest absorption band at 1340 cm^−1^ and a strong peak at 1053 cm^−1^, while the C-N stretching (-NH_2_) vibrations did not show any absorption. The other peaks of the reactant 3,5-diamino-1,2,4-triazole were clearly assigned [38].

Because of the -OH stretching of the new hindered phenolic group, a wide absorption band developed at 3430 cm^−1^. An absorption at 1428 cm^−1^ was seen for C-H bending, whereas the classic asymmetric C-H stretching peaks emerged at 2968 cm^−1^. In the novel synthesized material TZAB, the -N-H stretching (primary amine group) absorption bands at 3400 and 3100 cm^−1^ disappeared, while the -C=N stretching (imine) exocyclic double bond peak formed at 1661 cm^−1^ [39]. It was confirmed that the intermediate TZAB had the correct structure by observing the absorption band at 1093 cm^−1^ due to the C-N stretching vibration as well as other characteristic peaks, as presented in Scheme 1.

**Figure 2:**
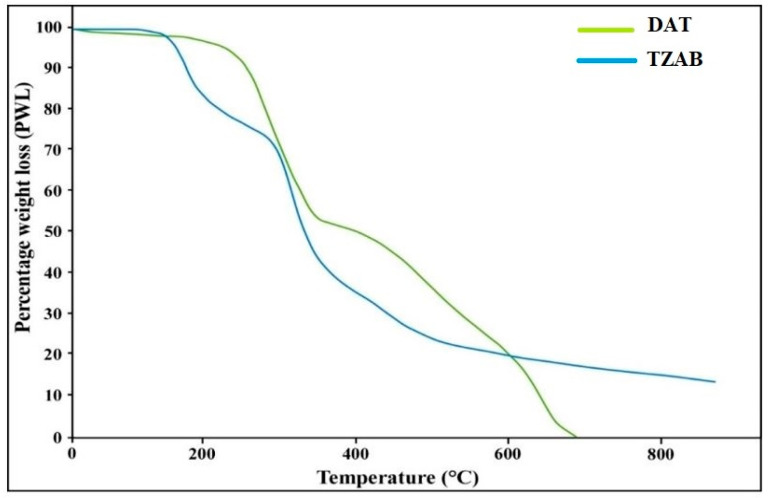
TGA curve of 3,5-diaminotriazole (DAT) and triazole Schiff base (TZAB).

The thermogravimetric analysis of the prepared adsorbent is presented in Figure 2, which shows that the imine coupling of 3,5-diamino-1,2,4-triazole (DAT) with 4-hydroxybutan-2-one was effective. The thermal analysis curve demonstrated that the adsorbent TZAB was less durable than the reactant DAT. The first temperature of degradation for DAT was observed at 275 °C, while the TZAB began to degrade at 188 °C. The DAT’s second degradation temperature was observed at 560 °C, and it was completely degraded at 712 °C. The second and third decomposition temperatures of the TZAB were 325 °C and 562 °C, respectively. Hence, Schiff coupling of triazole with a 4-hydroxybutan-2-one molecule decreased the heat stability.

1H-NMR (400.15 MHz, DMSO-d6, 25 °C, TMS) δ, ppm: 2.69 (d, 3H, J = 6.54 Hz, CH3-CH- methyl group), 3.07 (m, 2H, J = 5.79 Hz, -CH_2_-CH_2_OH methylene protons), 3.66 (m, 2H, J = 5.79 Hz, -CH_2_-CH_2_OH methylene protons), 4.73 (t, 1H, J = 5.5 Hz, -OH group), 6 (s, 1H, -NH group). 1H-NMR spectroscopy with an energy of 400.15 MHZ and DMSO-d6 as a diluent is an efficient and suitable instrument that provides substantial information about protons in the synthesized TZAB derv. ligand and can be used to assist with structural characterization.

**Figure 3:**
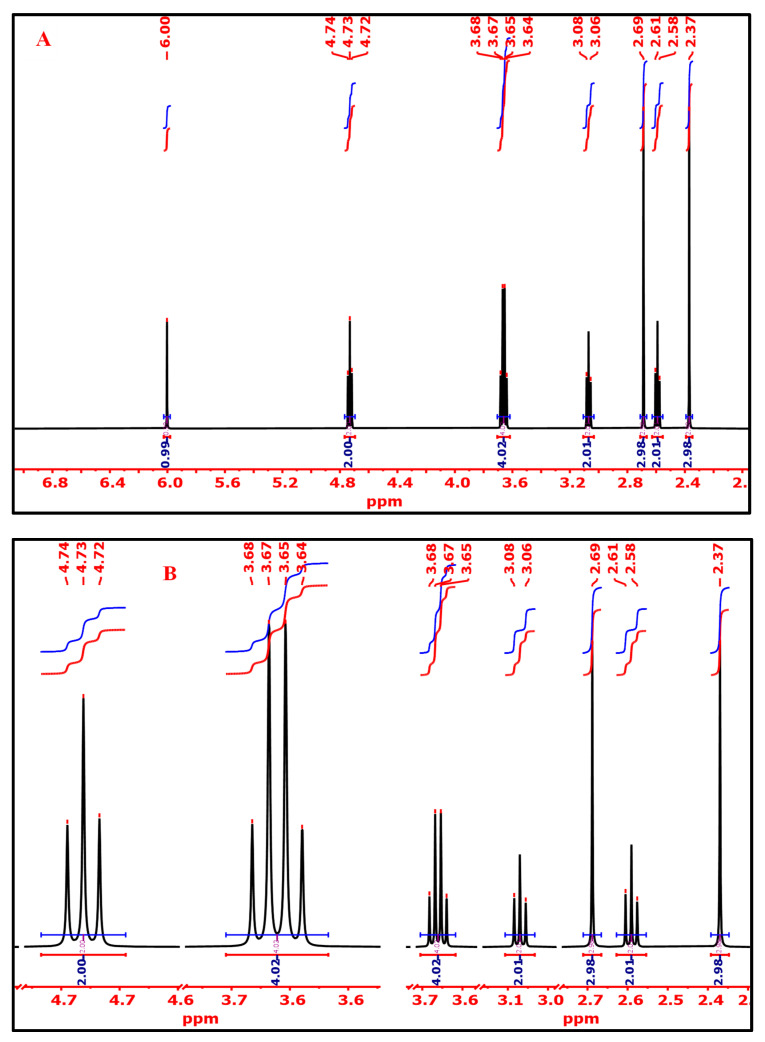
(**A**) Characterization of TZAB ligand by ^1^H-NMR Spectrometry. (**B**) Clarification of the ^1^H-NMR Spectrometry of TZAB ligand.

The major (δ ppm) assignments were performed at six ppm, which corresponds to the more deshielded -NH group protons. It was noted that the assignment of the -OH protons (δ = 4.73 ppm) was also deshielded, although less than the assignment of the -NH protons (δ = 6 ppm). The twin assignments of the methylene-group protons were detected at chemical shifts of 3.07 and 3.66 ppm, but the methyl group protons appeared to be significantly shielded compared to the methylene protons at 2.69 ppm. The characterization of the TZAB derv. ligand that occurred by using 1H-NMR spectroscopy is presented in Figure 3A,B.

**Figure 4:**
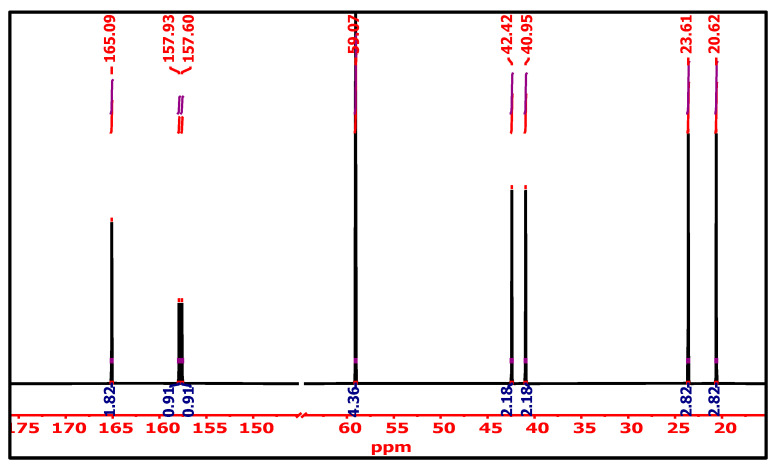
Characterization of TZAB ligand by ^13^C-NMR Spectrometry.

^13^C-NMR (100.01 MHz, DMSO-d6, 25 °C, TMS) δ, ppm: 20.6–23.6 (s, J = 4.1 Hz, -CH3), 59.1 (s, J = 2.2 Hz, -CH_2_-OH), 40.9–42.4 (s, J = 4.1 Hz, -CH_2_-CH_2_-OH), 165.1 (s, J = 5.2 Hz, -CH=N-), 157.9 (s, J = 0.6 Hz, -NH-C=N- of triazole ring). A 13C-NMR analysis associated with the energy of 100.01 MHZ and DMSO-d6 as a dilution agent is a dynamic tool which provides important data about the quantity of the carbon atoms in the synthesized ligand. The chief δ (ppm) performed about 20.6–23.6 ppm, which is associated with the more shielded methyl carbon. It was noticed that the methylene carbon attached to the hydroxyl group was further deshielded (59.1 ppm) than the next methylene carbon (40.9–42.4 ppm). The distinct assignment of the imine group carbon indicated that it was additionally deshielded and associated with a chemical shift of 165.1 ppm. The imine carbon of the triazole ring was established and gave a deshielded value of 157.9 ppm. The specification of the TZAB ligand by using 13C-NMR is shown in Figure 4.

**Figure 5:**
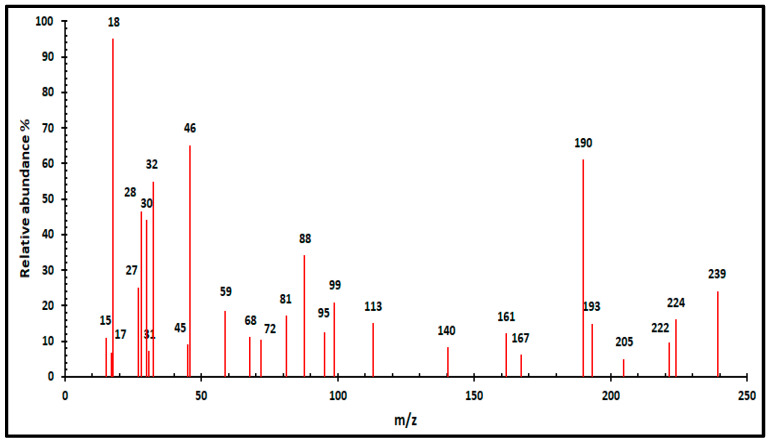
Characterization of TZAB ligand by mass Spectrometry.

GC/MS (EI, 30 eV), *m*/*z* (% rel): [m/e]+ of 239, 88, 99, 18, 32, 46, 15, 30, 27, and 28. Anal. Calc. for C_10_H_17_N_5_O_2_ (239.28 g/mol): C, 50.20; H, 7.16; N, 29.27; O, 13.37. Found: C, 50.18; H, 7.15; N, 29.25; O, 13.39. Gas chromatography associated with a Mass Spectrometer (GC/MS) is another important and strong technique used to determine different parameters, namely, the chemical formula, more stable fragment [*m*/*z*]+, and purity. Figure 5 shows a GC/MS analysis of the triazole derv. Specific patterns of significant fragmentation were observed and were related to the synthetically produced TZAB derv. chelating ligand; for example, C_4_H_8_O_2_ with a molecular weight (M.W) of 88 and a relative abundance of 34, which would be associated with 4-hydroxybutan-2-one, and C_2_H_5_N_5_ with a M.W of 99 and a relative frequency of 21, which is connected to 1,2,4-triazole-3,5-diamine moiety.

The water-releasing [H_2_O]˙ fragment had a M·W of 18 and a relative abundance of 95. The methanol-releasing [CH_3_OH]˙ fragment had a M.W of 32 and a relative abundance of 55. The ethanol-releasing [C_2_H_5_OH]˙ fragment had a M.W of 46 and a relative abundance of 65. The [CH_3_]˙ fragment had a M.W of 15 and an abundance ratio of 11. The [C_2_H_6_]˙ fragment had a M·W of 30 and a relative abundance of 44. The [HCN]˙ fragment had a M·W of 27 and a relative abundance of 25, which indicates the release of hydrogen cyanide gas. The [N_2_]˙ fragment had a M.W of 28 and a relative abundance of 46, which signifies the release of nitrogen gas. The molecular ion peak of the triazole derv. chelating ligand was identified at 239 with a relative abundance of 24. The overall analysis ensured a successful ligand synthesis.

### 3.2. The Factors Affecting the Adsorption of Cobalt

#### 3.2.1. Effect of pH

**Figure 6:**
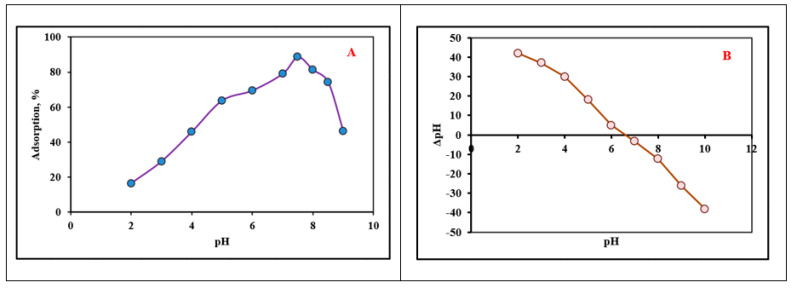
(**A**) Effect of pH on the removal of cobalt by TZAB; (**B**) pH point of zero charges of TZAB, [Co^+2^] = 250 mg⋅L^−1^, 0.1 g/20 mL TZAB, 30 min contact time

The pH variation is a significant factor in the adsorption process because of the hydrogen (H^+^) and hydroxyl (OH^−^) ions. This component can ensure the separation of the presented metal ions such as Co^2+^ and alter the surface chemistry of the chosen sorbent. Figure 6A illustrates the effect of the pH change on the Co^2+^ adsorption efficiency. The pH effect was investigated by varying it in the range from 2 to 10 for a Co^2+^ concentration of 250 mg/L.

The adsorbent’s surface charge and cobalt (II) species fluctuated when the solution pH changed. Figure 6B illustrates the principal species of cobalt (II) at pH 4–13, which would include Co^2+^, Co(OH)^+^, Co(OH)_2_, and Co(OH)^3−^. The dominating species was Co^2+^, and cobalt (II) was mostly eliminated via the adsorption reaction. The influence of pH on Co^2+^ sorption was calculated at pH values ranging from 2 and 9.

It was noted that when a pH of 7.5 was reached, the TZAB material showed an outstanding adsorption capacity towards Co^2+^ due to the protonation of the group reducing and the non-protonated groups being able to bind more Co^2+^. Low cobalt-ion adsorption was observed on the adsorbents in acidic and neutral environments. Because of the shift in potential at the adsorbent’s surface, the adsorption capacity rose dramatically, and the surface of the protonation turned cationic. In solutions with a pH better than pH_zpc_, the surface is negatively charged, making it attractive to cationic ions. The adsorbent’s surface can gradually transform from positive to negative as the pH value increases from 3.0 to 10.0. Cobalt ion sorption is improved by an adsorbent with a more negative potential. A pH of 7.5 was selected for the highest adsorption of cobalt by using the TZAB.

#### 3.2.2. Effect of Sorbent Dose

**Figure 7:**
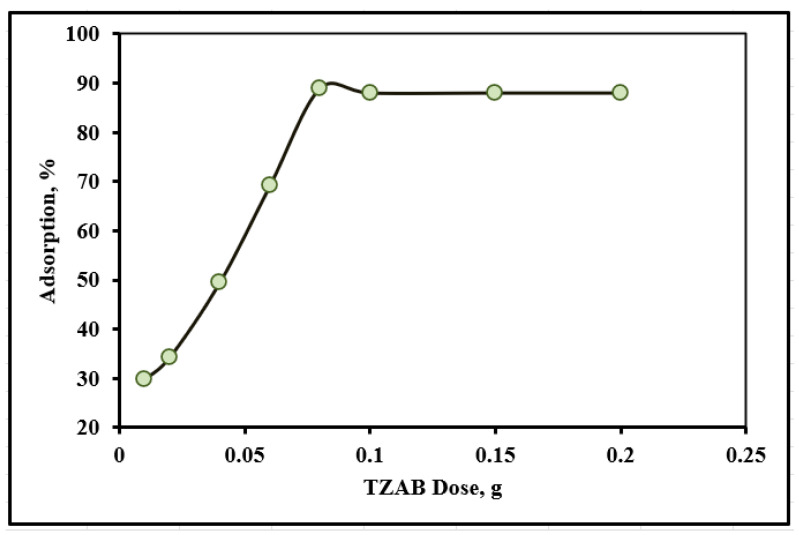
Influence of TZAB dose on Co^+2^ adsorption, [Co^+2^] = 250 mg⋅L^−1^, 7.5 pH, 30 min contact time.

The dosage of the sorbent has a significant effect on the practical application of the sorbent for the metal ion adsorption process [40,41]. The effect of the adsorbent dose on the Co^+2^ adsorption process was examined by employing variable amounts of the TZAB ranging from 0.01 to 1 g, which were added to 20 mL of the Co^+2^ solution with a 250 ppm concentration at an ambient temperature (25 °C), 7.5 pH, and contact time of 1 hr. The results obtained are presented in Figure 7. It was noted that the percent removal of Co^+2^ improved from 29.76 to 88.93% when the sorbent dose increased from 0.01 to 0.08 g. This can be attributed to the fact that the increase in the TZAB dose resulted in more active sites that were available for sorption at the same concentration of Co^+2^, so at 0.08 g of the TZAB, all the active sites were occupied and any excess TZAB dose addition did not affect the adsorption capability of the TZAB, as presented in Figure 7.

#### 3.2.3. Effect of Contact Time

**Figure 8:**
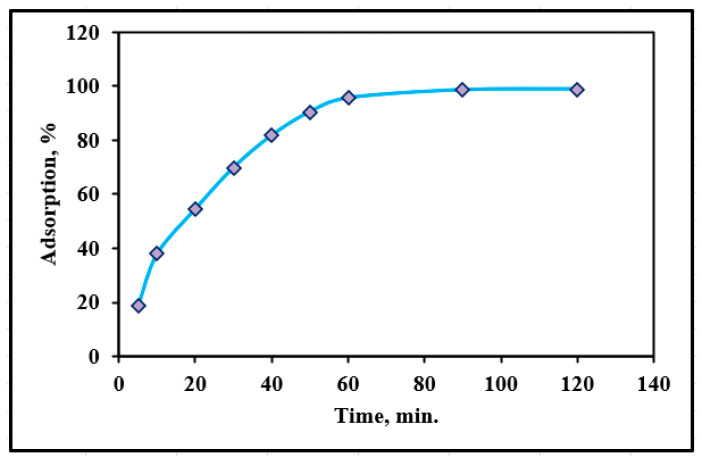
Effect of contact time upon the sorption of Co^+2^ by using TZAB adsorbent, [Co^+2^] ≈ 500 mg·L^−1^, dose of 0.08 g, pH 7.5, T = 25 °C.

The effect of the contact time on the Co^+2^ adsorption process was examined by changing the contact time in the range from 1 to 120 min at 0.08 g of a TZAB dose at 25 °C and 7.5 pH. The obtained results are presented in Figure 8. The adsorption process evolved over time via numerous steps of surface diffusion, penetration, and adsorption equilibrium. It was noted that the adsorption quantity increased rapidly over the first 30 min and reached 102.12 mg/g after 40 min. Subsequently, the sorption quantity gradually increased from 45 to 90 min and finally reached 122.87 mg/g at 90 min. After extending the contact time to 90 min, the TZAB adsorption capacity reached the equilibrium state, and nearly no further change was noted.

### 3.3. Kinetics of Co^+2^ Adsorption

The pseudo first-order model and pseudo second-order model (Equations (3) and (4)) were utilized to examine the adsorption kinetics of the TZAB [42].

(3) $$\ln\left(Q_{e}-Q_{t}\right)=\ln Q_{e}-k_{1}t$$

(4) $$\frac{t}{Q_{t}}=\frac{1}{k_{2}Q_{e}{}^{2}}+\frac{t}{Q_{e}}$$

where Ce (mg·L^−1^) represents the equilibrium concentration of Co^+2^; Q_t_ and Q_e_ (mg·g^−1^) represent the amount of cobalt adsorbed by the sorbents at time t and after reaching equilibrium, respectively; and k_1_ (min^−1^) and k_2_ (mg·g^−1^) represent the rate constants of the pseudo first- and second-order kinetics models.

**Figure 9:**
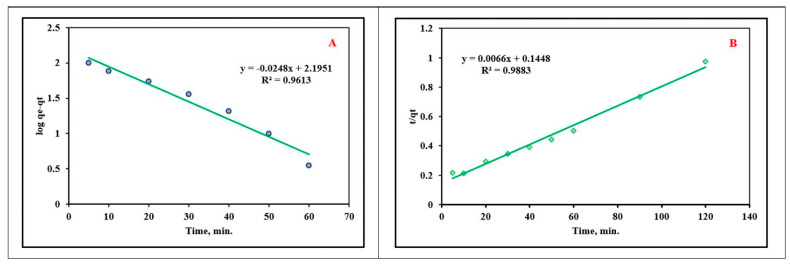
Fitting curve of (**A**) pseudo first-order model and (**B**) pseudo second-order model (Co ≈ 500 mg⋅L^−1^; dose = 0.08 g⋅L^−1^; pH = 7.5; and T = 25 °C).

It was noted that the TZAB adsorption process clearly fit the pseudo second-order kinetic model (R^2^ = 0.9983) more faithfully than that of the pseudo first-order kinetic equation (R^2^ = 0.9613), as presented in Table 1 and Figure 9, which suggests that the TZAB adsorption of Co^+2^ may depend mainly on the chemisorption process [43,44]. In the meantime, it can be concluded that the TZAB had a large number of active sites for the absorption of Co^+2^.

#### 3.3.1. Influence of Ionic Strength

**Figure 10:**
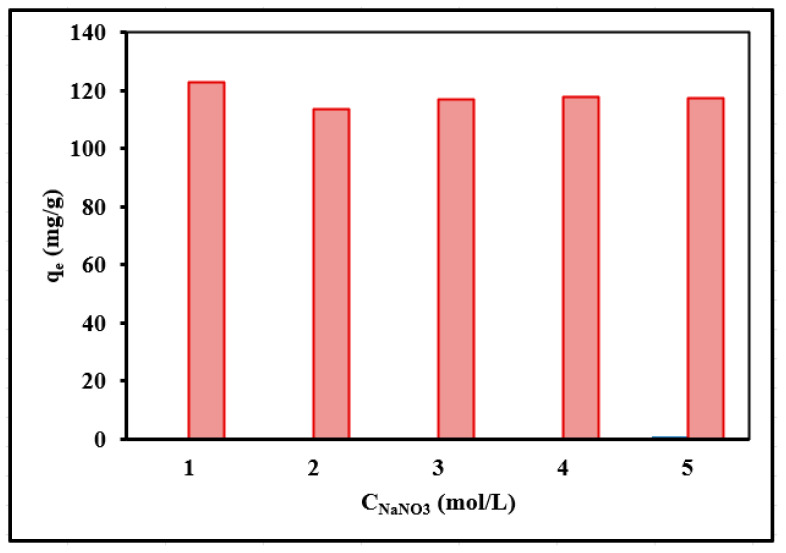
Effect of ionic strength on the sorption capacity of Co^+2^ on TZAB (C0 ≈ 500 mg·L^−1^; pH = 7.5; t = 90 min; and T = 25 °C).

The influence of the ionic strength on the cobalt adsorption behaviour can be utilized to differentiate the sort or type of complex formation between the sorbent and adsorbate surface. If the ionic strength has no effect on the sorption process, the adsorbent and adsorbate will form an inner surface complex. If the ionic strength increases, the adsorbent and adsorbate will construct an outer surface complex. The adsorption experiment was conducted with various NaNO_3_ concentrations (≈0, 0.001, 0.01, 0.1, and 1 mol/L). It was noted that the TZAB adsorption capacity to adsorb Co^+2^ was not sensitive to changes in the NaNO_3_ concentration, as shown in Figure 10. The sorption procedure of the manufactured sample was independent of ionic strength, and complexation on the inner spherical surface (chemical adsorption) instead of the outer spherical surface (physical adsorption) had a larger effect on the sorption of Co^+2^ on the TZAB [45]. The kinetic effect of the adsorption was evidenced by the experimental results. In conclusion, the TZAB adsorption followed the pseudo second-order model, which is controlled by chemical adsorption.

#### 3.3.2. Adsorption Isotherms and Thermodynamic Study

**Figure 11:**
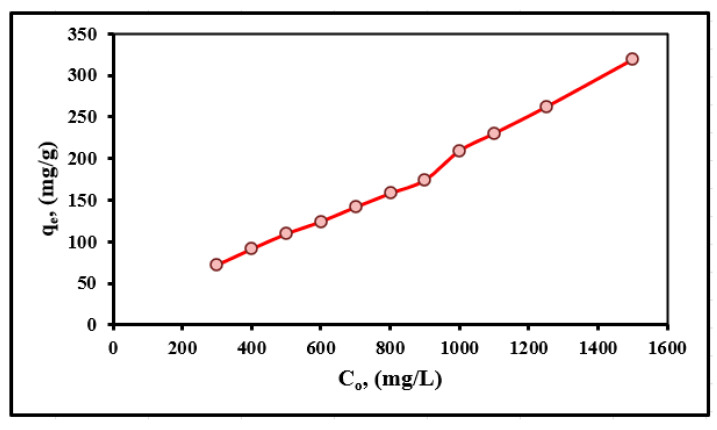
Effect of cobalt concentration in solution upon the adsorption process of TZAB (pH = 7.5; dose of 0.08 g; t = 90 min; and T = 25 °C).

As is well known, adsorbents are most characterized by their sorption capability and removal amount. The influence of different starting concentrations on the adsorption capability and removal amount of the synthesized sorbent was investigated in a sequence of experiments performed for this study. The adsorption capability increased and the TZAB removal rate decreased as the cobalt concentration increased, as shown in Figure 11. After 90 min of operation in the solution at pH = 7.5, the TZAB’s adsorption ability increased from 24.5 mg·g^−1^ to 319.7 mg·g^−1^. However, as the Co^+2^ concentration rose from 100 mg·L^−1^ to 1500 mg·L^−1^, the eliminate rate dropped from 99.33% to 83.9% after being immersed in the solution for 90 min at a pH of 7.5. The reduction in the extraction efficiency can be attributed to the scientific fact that the adsorbent dose remained unchanged, which means that the binding sites of the TZAB are limited. It is theorized that the reduction in the removal rate was due to the steady supply of the adsorbent. So, the TZAB had limited binding sites. Therefore, the ligand bound to Co^+2^ with more adsorption sites, which led to a higher concentration in the remaining cobalt ions as the Co^+2^ concentration rose.

**Figure 12:**
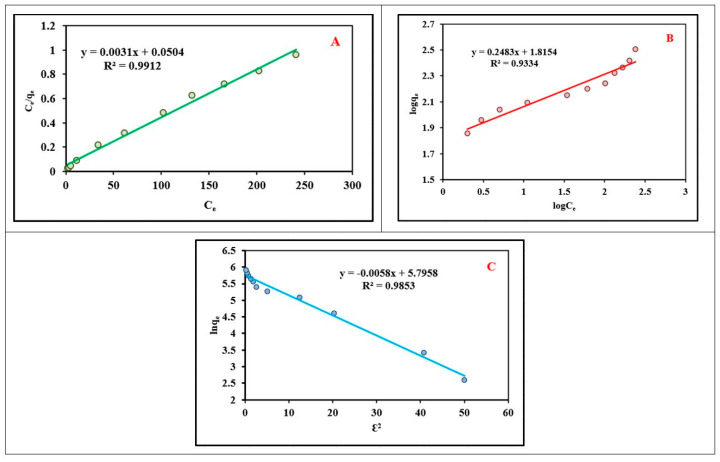
(**A**) Langmuir isotherm model, (**B**) Freundlich isotherm model, (**C**) Dubinin–Radushkevich model applied to determine Co^+2^ ions sorption by TZAB.

Three different adsorption isotherm models (Equations (5)–(12)) were used to model the adsorption process. The Langmuir and Freundlich isotherms and the D-R isotherm are presented in Table 2 and Figure 12. The Langmuir isotherm model is expressed in Equation (5):

(5) $$q_{e}=\frac{Q_{o}bC_{e}}{\left(1+bC_{e}\right)}$$

*C_e_* is the concentration of Co^+2^ in the solution (mg·dm^−3^) at equilibrium, and *q_e_* is the concentration of the solid-phase adsorbate (mg·g^−1^). Qo is the theoretical monolayer adsorption capacity (mg·g^−1^), and b is the adsorption energy (dm^3^·mg^−1^). The linear form of the Langmuir equation is given by Equation (6) [46]:

(6) $$\frac{C_{e}}{q_{e}}=\frac{C_{e}}{q_{m}}+\frac{1}{q_{m}K_{l}}$$

Using the linear plot of *C_e_*/*q_e_* vs. *C_e_* in Figure 12A, the values for *Q_o_* and *K_l_*, the Langmuir constants, were determined to be 322.58 mg·g^−1^ and 0.0615 dm^3^·mg^−1^, correspondingly (Table 2). A dimensionless constant, the equilibrium factors controlling R_L_ [47] describes the fundamental features of the Langmuir isotherm equation given by Equation (7):

(7) $$R_{L}=\frac{1}{\left(1+bC_{o}\right)}$$

The form of the isotherm is specified by the *R_L_* value, where *b* is the Langmuir constant and Co is the initial concentration (mg·g^−1^). For adsorption, the *R_L_* values between zero and one are optimal. All the concentrations of Co^+2^ studied had RL values between 0.0107 and 0.0514. The formula for the Freundlich isotherm is expressed in Equation (8) and its logarithmic form is expressed in Equation (9) [47]:

(8) $$q_{e}=k_{f}\;log\;C_{e}^{1/n}$$

(9) $$log\;q_{e}=log\;k_{f}+\frac{1}{n}log\;C_{e}$$

where *k_f_* is the adsorption capacity and *n* is the adsorption intensity. Figure 12B is a linear plot of log *q_e_* versus log *C_e_*, and the Freundlich constants and *k_f_* and *n* values of the adsorption of Co^+2^ by the TZAB are listed in Table 2. The form of the Dubinin–Radushkevich isotherm is expressed in Equation (10), and the linear Dubinin–Radushkevich (D-R) isotherm is expressed in Equation (11) [48,49,50]:

(10) $$q_{e}=q_{m}e^{-B\varepsilon^{2}}$$

(11) $$ln\;q_{e}=ln\;q_{m}-B\varepsilon^{2}$$

where *q_m_* is the theoretical saturation capacity in mol·g^−1^; β is the constant proportional to the average free energy of adsorption per mole of the adsorbate per mole mol^2^·J^−2^; and *ε* is the Polanyi potential proportional to the equilibrium concentration, which can be calculated by using Equation (12):

(12) $$\varepsilon =RTln(1+\frac{1}{C_{e}})$$

where *C_e_* is the equilibrium adsorbate concentration in the solution (mol·L^−1^), T (°K) is the absolute temperature, and R is the constant of universal gas (8.314 J mol^−1^·K^−1^). From the linear plot of *ln q_e_* vs. *ε*^2^, the D-R constants *q_m_* and *β* can be derived, which are presented in Table 2. In order to approximate the mean free energy E (kJ·mol^−1^) of adsorption per molecule of the adsorbate during its transfer from infinity in the solution to the surface of the solid, the used constant can be calculated by using Equation (13):

(13) $$E_{D}=\frac{1}{{\left(2\beta \right)}^{1/2}}$$

The value of this parameter specifies whether the sorption mechanism is physical or an ion exchange. Adsorption proceeds via an ion exchange for ED values ranging from 8 to 16 kJ mol^−1^ and is of a physical nature for ED values below 8 kJ mol^−1^ [51,52]. Adsorption in this study had a mean free energy of 8.01 kJ mol^−1^, which is consistent with an ion-exchange process, as shown in Figure 12C. Table 2 displays the correlation parameters, which showed that the Langmuir equation had a stronger correlation with the adsorption process than the Freundlich equation, despite the latter having a higher correlation coefficient (R^2^). The findings demonstrated that monolayer adsorption processes were occurring.

#### 3.3.3. Effect of Adsorption Temperature

**Figure 13:**
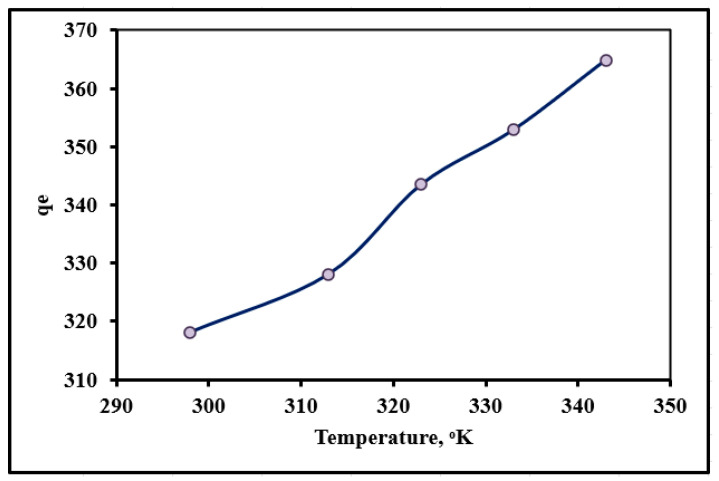
The effect of temperature on the adsorption of Co^+2^ on TZAB.

The effect of temperature on Co^+2^ ions sorption when using the TZAB adsorbent was studied at 0.08 g of TZAB that was added to 1500 ppm of Co^+2^ at pH 7.5 with a 90 min contact time in the temperature range from 25 °C to 70 °C. The obtained results are presented in Figure 13, which demonstrate that increasing the temperature led to a reduction in the uptake capacity of Co^+2^ by the TZAB adsorbent, indicating that temperature had a positive influence on the sorption of Co^+2^ ions by the TZAB and that the uptake was an endothermic process.

#### 3.3.4. Thermodynamic Investigation

To investigate the thermodynamic performance of the Co^+2^ ion sorption reaction system on the TZAB, enthalpy (ΔH), Gibbs free energy (ΔG), and entropy (ΔS) were calculated. These thermodynamic parameters were calculated by using Equations (14) and (15) [53,54,55]:

(14) $$LogKd=\frac{\Delta S}{2.303R}-\frac{\Delta H}{2.303RT}$$

∆G° = ∆H° − T∆S°(15)

where *K* is the reaction constant, *T* (°K) is the absolute temperature, and *R* is the universal constant of gas (8.314 J/mol·K).

**Figure 14:**
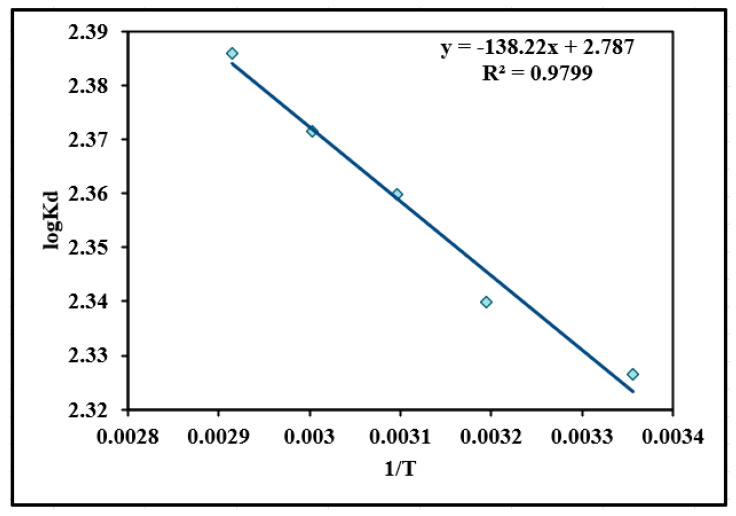
Plot of log Kd vs. 1/T for the adsorption of Co^+2^ using TZAB.

Table 3 displays the thermodynamic factors controlling the adsorption processes that were determined from experiments conducted at temperatures of 298, 313, 323, 333, and 343 °K. The values of Δ*S* and Δ*H* could be derived from the slope of the intersection between the Log Kd against the 1/T plot, as shown in Figure 14. As the obtained value of Δ*H* was positive, this means that the sorption of Co^+2^ onto the TZAB sorbent was endothermic. The sorption of Co^+2^ by the TZAB led to a rise in the randomness of the solid–liquid interface due to the positive ΔS value. Since ΔG was negative, this means that Co^+2^ adsorption onto the TZAB adsorbent occurred spontaneously.

### 3.4. Selectivity of TZAB for Metal Ions

**Figure 15:**
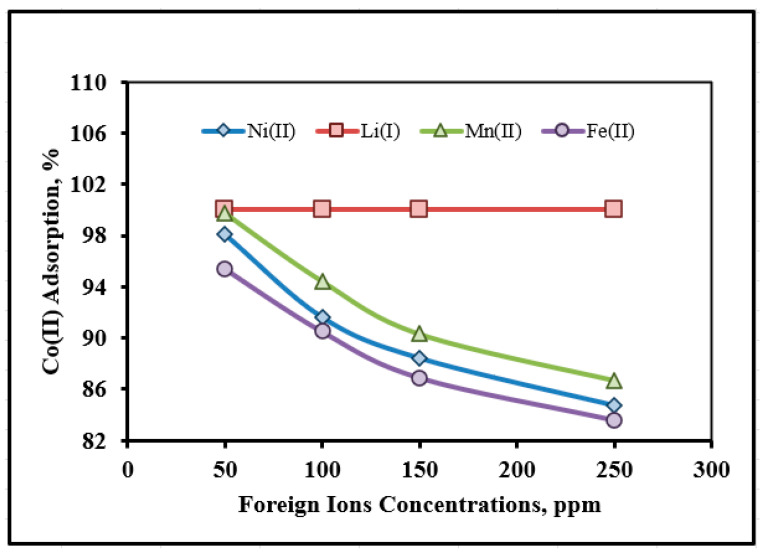
Impact of foreign ions on the sorption efficiency of Co^+2^ by using TZAB adsorbent.

The selectivity of the used adsorbent was investigated by studying the adsorption behavior of Co^+2^ in the presence of metal ions in the solution. The effect of the presence of varying concentrations of Mn(II), Ni(II), Fe(II), and Li(I) in the range from 10 to 250 mg/L on the sorption efficiency of Co^+2^ when using the TZAB adsorbent was investigated, which explained the Co^+2^ competition for adsorption sites in the presence of other metal ions. The obtained results presented in Figure 15 indicate that the selectivity of the TZAB was not affected by the presence of Li(I) ions, was a little affected by the presence of Mn(II) and Ni(II), and was moderately affected by the presence of Fe(II), which resulted in a reduction in the adsorption efficiency to 82% when the concentration of iron was 250 ppm. This can be attributed to the ability of the TZAB adsorbent to adsorb foreign cations through a chelation mechanism.

### 3.5. Elution and Regeneration of TZAB Adsorbent

**Figure 16:**
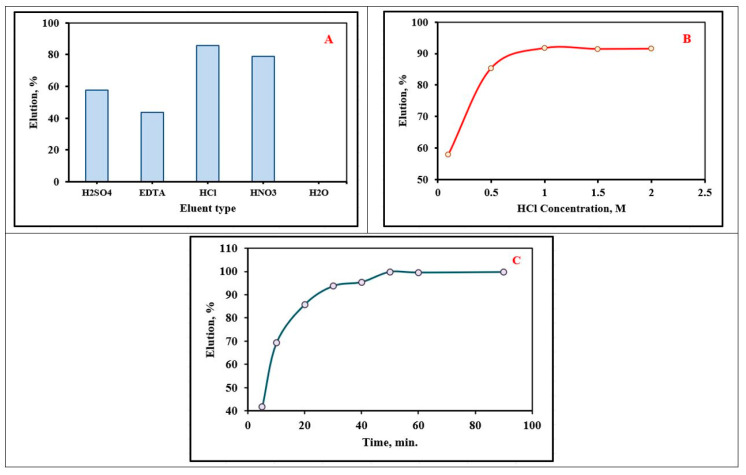
(**A**) Elution of Co^+2^ from TZAB surface by using different eluting solutions and (**B**) using different concentrations of HCl solution (**C**) at different elution times.

The absorbed Co^+2^ on the surface of the TZAB was recovered from the TZAB by utilizing several eluents, namely 0.5 M solutions of different acids (HCl, H_2_SO_4_, EDTA, and HNO_3_). The leaching of the adsorbed Co^+2^ ion from the sorbents was also tested by using deionized water as a control. The highest Co^+2^ ion recovery of 85.72% from the TZAB was obtained by using a 0.5 M HCl solution preceded by HNO_3_, H_2_SO_4_, and EDTA solutions, as presented in Figure 16. Figure 16A shows that the most efficient eluent type for the recovery of Co^+2^ was HCl, which was capable of recovering 85.72% of the loaded Co^+2^. The effect of the HCl concentration on the percent recovery of Co^+2^ was investigated by applying different concentrations of HCl in the range from 0.1 to 2 M, and the obtained results are presented in Figure 16B. The results showed that using 1 M HCl could result in the recovery of 91.7% of the loaded Co^+2^ from the TZAB adsorbent. Finally, the effect of the elution time on the Co^+2^ recovery from the loaded TZAB adsorbent was investigated by varying the elution time in the range from 5 to 90 min, and the obtained results are presented in Figure 16C. It is clear from Figure 16C that 50 min was sufficient to recover almost all of the Co^+2^ on the loaded TZAB. More than 99% of the loaded Co^+2^ was recovered at a 50 min elution time.

### 3.6. Durability of TBAZ for the Sorption of Co^+2^ Ions

**Figure 17:**
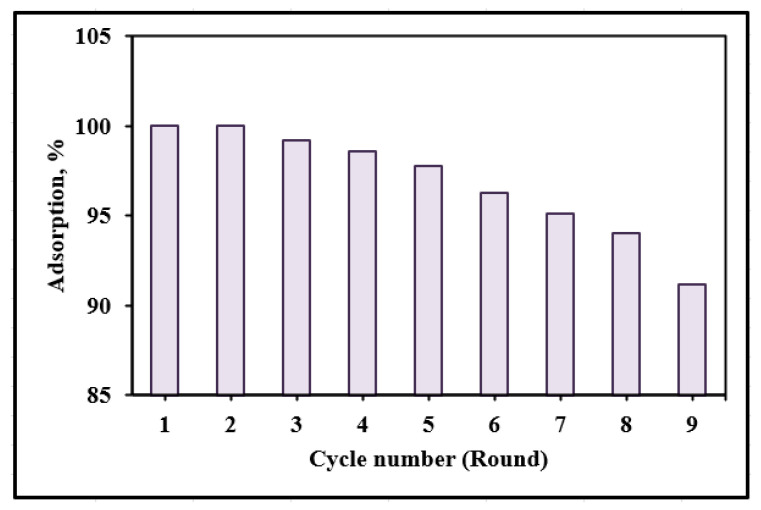
Recycling of TZAB for the adsorption of Co^+2^ ions.

The ability to regenerate ion-imprinted polymers is crucial to their utility in the real world. Hence, TZAB sorption–elution tests were studied. In this case, 20 mL of a Co^+2^ solution was added to 0.08 g of adsorbent and agitated for 90 min at 70 °C before the Co^+2^ concentration was determined. By using an a1M HCl solution, the TZAB was regenerated. The obtained results of the TZAB adsorbent regeneration are shown in Figure 17. It was noted that after five cycles of recycling, the TZAB still had a high uptake capacity, with an efficiency of over 97%. This confirms the actual utilization of the TZAB adsorbents in the wastewater treatment process. Based on the obtained results of the current investigation, the TZAB shows promising potential for adsorbent utilization for Co^+2^ separation in a real wastewater sample.

In order to give more attention to the advantages and the importance of the synthesized adsorbent, a comparison between different adsorbents prepared for the adsorption of Co^+2^ is presented in Table 4. It was noted that the prepared TZAB adsorbent had a maximum adsorption capacity of 319.7 mg·g^−1^ at the optimum operation conditions, which is higher than most other sorbents for which data are available.

**Scheme 2:**
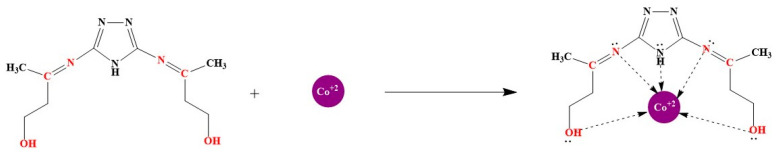
The proposed mechanism of interaction between Co^+2^ metal ions and TZAB adsorbent.

Scheme 2 presents the proposed possible chelation and adsorption mechanism for the Co^+2^–TZAB interaction, involving the imine’s lone pair of electrons and the long pairs of electrons found in hydroxyl groups.

### 3.7. Application of TZAB Adsorbent in Processing Recycled Li-Ion Batteries

In this section, the Li-ion batteries collected from laptops fitted with LiCoO_2_ (ICR) cathodes obtained from the secondary IT device market and repair shops are utilized for the extraction of Co^+2^ metal ions. The plastic container covering the cells was removed by hand sorting. To prevent the occurrence of a short circuit, the cells were first submerged in an electrolyte solution of 5% NaCl *w*/*v* for one day before being removed, were rinsed in deionized water, and were dried at 90 °C for 12 h. Then, a manual opening was carried out by cutting a cross section through the metal cap. After cutting away the steel casings of the cell, the contents of the cell were sorted into their different component parts: plastic, aluminum sheets of the cathodes, and copper sheets of the anodes. The powdered cathodic active material was easily removed from the aluminum sheets by heating them in the temperature range of 250 °C to 300 °C for 30 min. The recovered powder was milled to a mesh size of −200 μm before being sieved.

The leaching experiments were conducted by using sulfuric acid with a 2 M concentration at 60 °C for 60 min at a 40 g/L solid/liquid ratio and a 15% hydrogen peroxide concentration, which resulted in the extreme leaching efficiencies of the Co^+2^ and Li metal ions. Co^+2^ is more leachable with H_2_SO_4_ because hydrogen peroxide causes a reduction of Co from Co^+3^ to Co^+2^. In terms of stability, Co^+2^ is more stable than Co^+3^. Both Li and Co are leached even at the minimum sulfuric acid concentration [68]. The leaching process of LiCoO_2_ in the H_2_SO_4_ solution was carried out in accordance with Equation (16) [69]:

2LiCoO_2(s)_ + 3H_2_SO_4(aq)_ + H_2_O_2(aq)_ → 2CoSO_4(aq)_ + Li_2_SO_4(aq)_ + 4H_2_O_(g)_ + O_2(g)_ (16)

In order to separate the leachate solution from the insoluble residues, a La fil 400 vacuum filtration system was employed, and Whatman Grade GF/B filter paper (12.5 cm size) was employed for the filtering. ICP-OES was used to examine the metal value content of the Co and Li in the liquid samples, as shown in Table 5.

**Figure 18:**
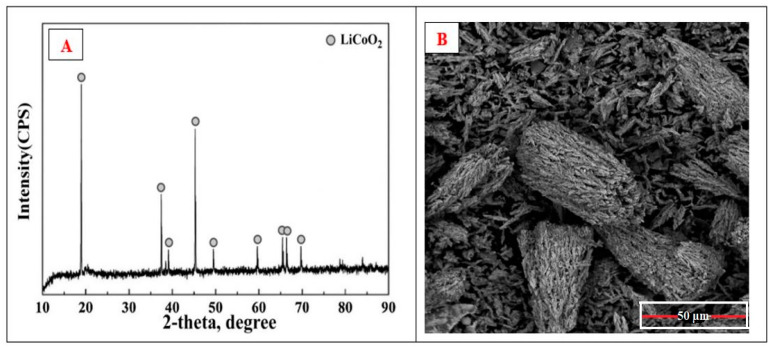
(**A**) SEM images of cathodic material LiCoO_2_ and (**B**) X-ray diffraction analysis of cathode electrode material.

The physical analysis of the black cathodic material by X-ray diffraction presented in Figure 18A revealed that the Li and Co metal ions were presented in the form of LiCoO_2_, which considered the principal source of the cathode active material. All the sharp peaks in the diffraction pattern referenced LiCoO_2_, and no additional peaks were visible. It was noted that the high crystallinity regenerated material formed because of the characteristic peak’s sharpness. A SEM analysis was also performed on the black powder. The particles had a fibrous crystallite shape, as observed on the deposit surface in Figure 18B [70]. Therefore, the LiCoO_2_ powders derived from the used LIBs cannot be used directly as active materials in cathode production unless they are first recovered and purified.

Ten grams of the spent black powder of the cathode was subjected to a leaching process by using 250 mL of 2 M sulfuric acid in the presence of 15% hydrogen peroxide at 60 °C for 60 min. The obtained leach liquor was filtrated, and the cobalt and lithium content were measured by using ICP-OS; the concentration of Co^+2^ was found to be 5612 ppm while the lithium concentration was 1091 ppm. The second step was applying the best controlling parameters that were optimized by using the TZAB adsorbent for the adsorption of Co^+2^ from the black powder leach liquor so that 1 g of the TZAB was speared on 250 mL of the spent batteries’ cathode leach liquor. The solution was adjusted at 7.5 pH (by using 1 M of NH_4_OH or 1 M H_2_SO_4_) and stirred for 60 min at 70 °C. It was noted that the maximum leaching efficiency of the Co^+2^ was 95%, and then the Co^+2^ loaded on the TZAB was subjected to an elution process by using a 1M HCl solution that was stirred for 50 min at 25 °C. The obtained liquor was subjected to precipitation by using ammonium oxalate and by adjusting the solution pH to 1.5 and stirring speed to 300 rpm for 60 min at 75 °C in accordance with Equation (17). It has been suggested that the following process can be used to recover cobalt from ammonium oxalate; after numerous washes and drying, the resulting solids were analyzed by using a SEM-EDX analysis:

Li^+^_(aq)_ + Co^+2^_(aq)_ + (NH_4_)_2_C_2_O_4(aq)_ → CoC_2_O_4(s)_ + Li^+^_(aq)_ + 2NH^+^_(aq)_ (17)

**Figure 19:**
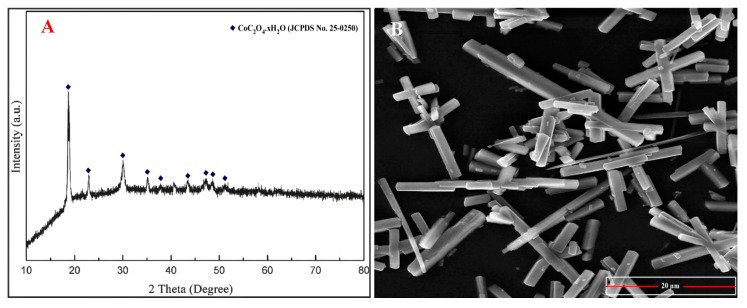
(**A**) XRD analysis and (**B**) SEM of the solid precipitation of cobaltous oxalate CoC_2_O_4_.

The recovered CoC_2_O_4_ from the extraction processes was characterized by using XRD and SEM-EDX analyses. The obtained XRD analysis is presented in Figure 19A, and micrographs of the resultant product are presented in Figure 19B.

Figure 19 illustrates the results of an X-ray diffraction (XRD) analysis on the crystal structure of the cobalt oxalate product. According to the pattern, all the diffraction peaks exhibited properties of the cobalt oxalate phase, which can be indexed to the orthorhombic phase of CoC2O4.H2O (JCPDS No. 25-0250) [71].

**Figure 20:**
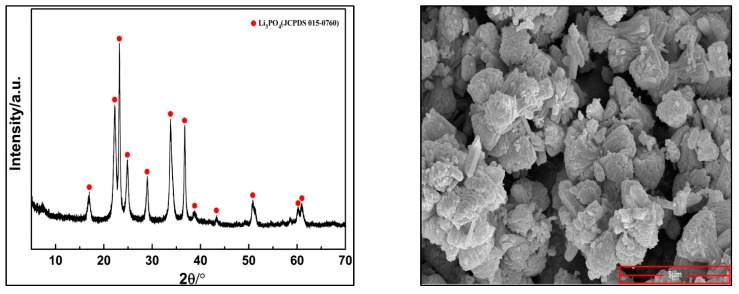
(**A**) XRD analysis and (**B**) SEM of the solid precipitate of lithium phosphate (Li_3_PO_4_).

The residual filtrates after separating CoC_2_O_4_ contained all lithium, which was subjected to precipitation by using phosphoric acid. Then, the pH of the leaching liquor was adjusted by using NaOH and heating the solution to 70 °C at a 300 rpm stirring speed for 60 min to produce pure Li_3_PO_4_ according to Equation (16). The obtained Li_3_PO_4_ precipitate was characterized by using XRD and SEM-EDX analyses, as presented in Figure 20A,B. The precipitation and purification procedure, which included multiple washes with distilled water, resulted in a relatively pure Li_3_PO_4_ product (with a purity of 98.3%). One possible reaction for lithium recovery is described in Equation (18):

3Li^+^_(aq)_ + H_2_PO_4_^−^_(aq)_ + 2NaOH_(aq)_ → Li_3_PO_4(s)_ + 2Na^+^_(aq)_ + 2H_2_O (18)

The XRD analysis of the white precipitate of lithium phosphate shows that the diffraction lines of the precipitated solid corresponded to Li_3_PO_4_ (JCPDS 015-0760), while the SEM analysis showed that lithium phosphate particles had an olivine structure with a diagonal length between 1 and 2 μm [42].

**Figure 21:**
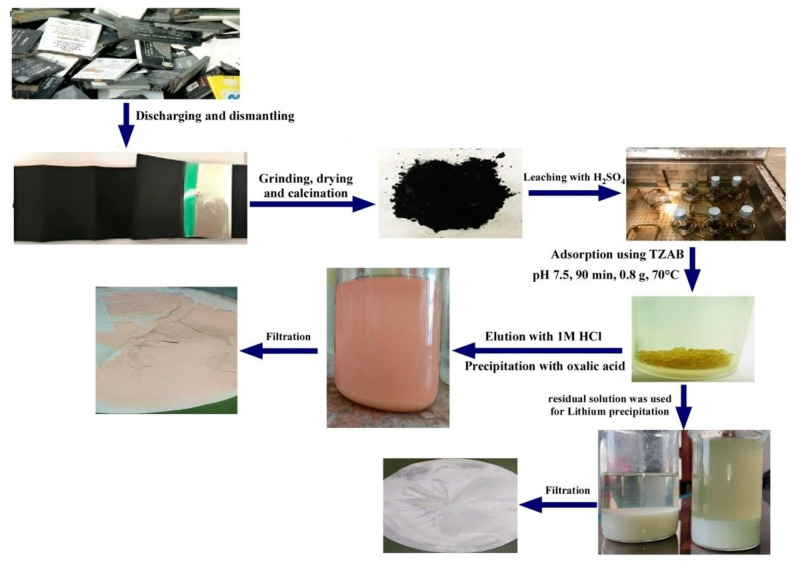
The proposed flowsheet of cobalt and lithium extraction from spent LIBs by using novel synthesized (TZAB) adsorbent.

The proposed flowsheet of the cobalt and lithium extraction from the spent LIBs by using a novel synthesized (TZAB) adsorbent is presented in Figure 21.

## 4. Conclusions

The global increasing interest in clean and green energy resources and environmental restrictions has led to the production of a huge number of energy storage batteries. One of the promising types of these batteries is LIBs, which have shown advanced operational and technological properties. Due to the significant increase in the consumption of lithium batteries, a huge number of discarded lithium batteries will be generated accordingly. The present study aimed to explore the processing of discarded LIBs by using ecofriendly and cost-effective processes to reduce the negative environmental effects and at the same time utilize them to produce valuable metals. The proposed technique presented here applies a novel synthesized solid-phase (TZAB) adsorbent in the hydrometallurgical processing operation to selectively remove cobalt and lithium from synthetic solutions and discarded LIBs.

The obtained results showed that the optimum conditions for the maximum recovery of Co(II) from synthetic solutions were as follow: C_0_ = 500 mg·L^−1^, dose of 0.08 g, pH 7.5, T = 25 °C, and reaction time = 90 min. The collected data from Langmuir’s isotherm and the adsorption processes of Co agreed with the data predicted by the D-R isotherm models, which showed that the adsorption of Co(II) onto TZAB appeared to be chemisorption, and the results were in good agreement with the Langmuir and D-R isotherm models. The experimental data on dynamics also fit the pseudo second-order kinetic equation nicely. According to the thermodynamic data, it was determined that the reaction behavior was spontaneous and endothermic.

The application of the novel synthesized adsorbent and the optimized adsorption conditions on the selected sample of discarded lithium-ion batteries led to the recovery of high-purity cobaltous oxalate and lithium phosphate. High-purity CoC_2_O_4_ and Li_3_PO_4_ were obtained with a purity of 95% and 98.3% and a percent recovery of 93.48% and 95.76%, respectively. The synthesized TZAB adsorbent is one of the highly recommended adsorbents due to its high adsorption capacity and cost effectiveness in comparison with other adsorbents. All this enhances its application in the processing of spent LIBs for the selective adsorption of Co(II) and lithium.

## Figures and Tables

**Table 1:** Kinetic parameters of Co^+2^ adsorption upon TZAB.

1st Order Kinetic				2nd Order Kinetic		
K_1_	q_(max)cal_	R^2^	q_max(exp)_	K_2_	q_(max)cal_	R^2^
0.0571	156.711	0.9613	123.1	0.0004	128.21	0.9883

**Table 2:** Factors of isotherms of different models for sorption of Co^+2^ ions using TZAB.

Kinetic Models	Parameters	
Langmuir isotherm	Equation	y = 0.0031x + 0.0504
	q_max_ (mg/g)	322.58
	K_1_	0.06151
	R^2^	0.9912
Freundlich isotherm	Equation	y = 0.2483x + 1.8154
	K_f_ (mg/g)	65.373
	1/n (mg min/g)	0.2483
	R^2^	0.9334
D-R isotherm	Equation	y = −0.0058x + 5.7958
	q_D_ (mg/g)	328.915
	B_D_ (Mo12/KJ2)	0.0058
	E_D_ (kj/mo^−1^)	9.28477
	R^2^	0.9541
Practical Capacity	q_exp_	319.7

**Table 3:** Thermodynamic parameters for Co^+2^ ions sorption by TZAB at different temperatures.

ΔH° (kJ mol^−1^)	ΔS° (J mol^−1^ K^−1^)	ΔG° (kJ mol^−1^)				
2.647	53.363	298 K	313 K	323 K	333 K	343 K
		−13.26	−14.06	−14.59	−15.12	−15.66

**Table 4:** Studying how various adsorbents compare favorably against one another when absorbing Co^+2.^

Adsorbent	mg·g^−1^	Reference
Phosphorylated MWCNT-cyclodextrin/silver-doped titania	7.81	[56]
PTU-MS silica	87.6	[57]
Imprinted CTS-GPTMS-PTW	22	[58]
MCC- MH composite	153.84	[59]
MCS/AC@UiO-66	44.5	[60]
Imprinted UiO-66-NH2	175	[61]
Sulfurized activated carbon	153.6	[62]
Imprinted silica	106	[63]
Natural vermiculite	49.5	[64]
Imprinted mesoporous silica	181.7	[65]
Magnesium hydroxide powders	125	[66]
Magnetite-alginate nanoparticles	33.6	[67]
Triazol Schiff base derivatives (TZAB)	319.7	Current study

**Table 5:** Chemical composition of the starting cathode material before leaching.

Metal	Li	Co	Al	Mn
Content (wt.%)	5.83	46.98	0.04	0.02
